# Integrated Proteomic and scRNA‐Seq Analysis Reveals Pyroptosis‐Related Subtypes in Lung Adenocarcinoma

**DOI:** 10.1111/crj.70210

**Published:** 2026-07-05

**Authors:** Tianchang Wei, Yongqi Wei, Weiqi Mao, Cuiping Zhang, Jian Xu, Chunlai Lu, Yuanlin Song, Xiaoyan Chen

**Affiliations:** ^1^ Department of Pulmonary Medicine, Shanghai Key Laboratory of Lung Inflammation and Injury Zhongshan Hospital, Fudan University Shanghai China; ^2^ Department of Thoracic Surgery Zhongshan Hospital, Fudan University Shanghai China; ^3^ Shanghai Institute of Infectious Disease and Biosecurity Shanghai China; ^4^ Shanghai Respiratory Research Institute Shanghai China; ^5^ Key Laboratory of Chemical Injury, Emergency and Critical Medicine of Shanghai Municipal Health Commission, Center of Emergency and Critical Medicine Jinshan Hospital of Fudan University Shanghai China

**Keywords:** lung adenocarcinoma, prognosis immune signatures, pyroptosis

## Abstract

**Background:**

Pyroptosis is a recently identified form of programmed cell death that plays an important role in cancer initiation and progression. However, the function of pyroptosis in lung adenocarcinoma (LUAD) remains unclear. The study integrated proteomic and single‐cell RNA sequence (scRNA‐seq) data to investigate the potential role of pyroptosis‐related proteins (PRPs) in the tumor microenvironment (TME) and their associations with tumor immunity, prognosis, and therapeutic response.

**Methods:**

A comprehensive proteomic analysis was conducted on 103 lung adenocarcinoma patients. We integrated scRNA‐seq data with bioinformatics analyses to evaluate prognostic and immunological characteristics. Unsupervised clustering based on the PRP expression identified three subtypes. We then used gene set enrichment analysis (GSEA) to assess biological functions. We applied TME and CIBERSORT algorithms to analyze immune cell infiltration. Next, we performed functional enrichment, immune infiltration, and cell–cell communication analyses using scRNA‐seq data grouped according to the proteomic subtypes. We constructed a PRP‐related prognostic signature using least absolute shrinkage and selection operator (LASSO)‐Cox regression analysis. Finally, we validated the expression and functional significance of selected proteins by immunohistochemistry, Western blotting, quantitative PCR, and in vitro loss‐of‐function assays.

**Results:**

We identified multiple PRPs that correlated with clinicopathologic characteristics, patient prognosis, and immune infiltration patterns in the TME. We further identified three distinct molecular subtypes, including lipid metabolism, signaling transduction, and immune activation. Among these subtypes, the lipid metabolism subtype showed the best prognosis. ScRNA‐seq analysis further supported the immune characteristics of three subtypes and revealed distinct cellular interaction networks and ligand‐receptor pairs. We also established and validated a PRP‐related prognostic score that effectively predicted overall survival in patients with LUAD. In addition, the PRP score was significantly associated with tumor immune status and sensitivity to chemotherapeutic agents.

**Conclusion:**

Our integrated analysis of proteomic and scRNA‐seq data demonstrated that PRPs are closely associated with the tumor‐immune‐stromal microenvironment, clinicopathological features, and prognosis in LUAD. These findings improve our understanding of the biological roles of PRPs in LUAD and may provide new strategies for prognostic evaluation and immunotherapy development.

## Graphical abstract

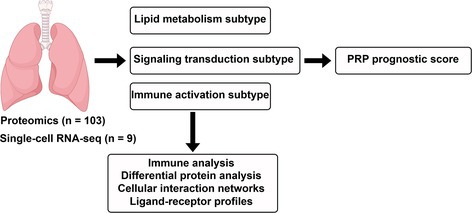

**Abbreviations**
AIC: Akaike information criterion
ANGPT: angiopoietins
ANXA1‐FPR1: annexin A1‐ fMet‐Leu‐Phe receptor
BAFF: B‐cell activating factor
CCLE: the Cancer Cell Line Encyclopedia
CDF: cumulative distribution function
DEPs: differentially expressed proteins
FGF: fibroblast growth factor
GSEA: gene set enrichment analysis
GSVA: gene sets for variant analysis
IC50: concentration in half
IHC: immunohistochemistry
LRP1: SP1‐LDL receptor‐related protein 1
LUAD: lung adenocarcinoma
MDK: midkine
NCL: neuronal ceroid lipofuscinoses
OS: overall survival
PRPs: pyroptosis‐related proteins
RFS: recurrence‐free survival
ROC: receiver operating characteristic curve
scRNA‐seq: single‐cell RNA sequence
SEMA3: class 3 semaphorin proteins
ssGSEA: the Single‐Sample Gene Set Enrichment Analysis
TME: tumor microenvironment
UGRP1: uteroglobin‐related protein 1

## 1 Introduction

Pyroptosis is a recently identified form of programmed cell death that relies on caspase‐triggered cleavage of gasdermin proteins following the recognition of ligands in the cytoplasm [1]. Members of the gasdermin family serve as the direct executors of pyroptosis. The main pathways of pyroptosis identified in recent studies include the caspase‐1/‐4/5/‐11‐mediated inflammatory‐dependent pathway and the caspase‐3/‐8‐associated inflammatory‐independent pathway [2]. Previous studies have demonstrated that pyroptosis is closely associated with the tumor immune microenvironment in several cancers, including breast cancer and colorectal cancer, thereby influencing tumor progression and patient prognosis [3, 4, 5].

Lung cancer remains one of the most common malignant tumors, resulting in 1 796 000 cancer‐related deaths per year worldwide [6, 7]. Lung adenocarcinoma (LUAD) is the predominant histologic subtype of lung cancer. Although therapeutic strategies for LUAD have improved substantially in recent years, including the application of immunotherapy, many patients still experience poor clinical outcomes. Tumor heterogeneity, resistance to chemotherapy and radiotherapy, and differences in tumor immunogenicity contribute to an unfavorable prognosis. The 5‐year survival rate of patients with advanced lung cancer ranges from 0% to 10%, whereas patients with early‐stage disease have a survival rate of approximately 60% [8, 9]. Therefore, identifying novel biomarkers and therapeutic targets for LUAD remains an urgent priority.

Accumulating evidence suggests that pyroptosis is closely associated with tumor development and progression. Pro‐inflammatory cytokines such as IL‐1*β* and IL‐18 can promote a microenvironment that supports tumorigenesis and progression [10]. Recent studies have also demonstrated extensive interactions between pyroptosis and the tumor immune microenvironment. In addition, pyroptosis may function as a form of immunogenic cell death and may enhance the efficacy of cancer immunotherapy [11]. In LUAD, previous studies have shown that increased TP53 expression suppresses tumor progression by inducing pyroptosis [12]. However, other studies have reported that pyroptosis may also contribute to chemotherapy resistance in LUAD [13]. These findings suggest that pyroptosis exerts complex and context‐dependent effects in LUAD. Therefore, further investigation of the molecular mechanisms underlying pyroptosis may provide new insights into LUAD diagnosis and treatment. In this study, we comprehensively analyzed the expression patterns of pyroptosis‐related proteins (PRPs) using proteomics data and further characterized the tumor immune landscape through scRNA‐seq analysis [14, 15]. We first classified 103 patients with LUAD into three distinct clusters based on PRP expression profiles. We then identified the biological and immunological characteristics of these subtypes based on differentially expressed proteins (DEPs). Furthermore, we established a DEP‐based prognostic scoring system to predict overall survival (OS) and evaluate the immune status of LUAD. This scoring system also enabled the prediction of patient prognosis, immunotherapy response, and sensitivity to chemotherapeutic agents. In addition, we validated the expression and functional roles of selected DEPs using immunohistochemistry in clinical specimens, as well as Western blotting, quantitative PCR, wound‐healing assays, and invasion assays in vitro. Finally, we performed comprehensive scRNA‐seq analyses to explore potential interactions between tumor cells and immune cells within the tumor microenvironment (TME).

## 2 Methods

### 2.1 Data Sources

Proteomic data and corresponding clinical information from two LUAD cohorts were obtained from previously published studies [14, 16]. We standardized protein expression intensities across the two cohorts before subsequent analyses. We downloaded the LUAD RNA‐seq dataset GSE41721 from the Gene Expression Omnibus (GEO) database (https://www.ncbi.nlm.nih.gov/geo/) for external validation. We normalized the raw FPKM data before analysis. We obtained scRNA‐seq data from the GEO database (GSE171145), which included 40 799 cells from nine LUAD samples.

Cells with fewer than 500 detected genes, mitochondrial gene content > 15%, or hemoglobin gene content > 5% were excluded during quality control. Gene expression data were normalized and scaled using the “Seurat” R package. Highly variable genes were identified using the “FindVariableFeature” function. Principal component analysis (PCA) was subsequently performed for dimensionality reduction, followed by unsupervised clustering and Uniform Manifold Approximation and Projection (UMAP) visualization. Tumor cells were identified using the “infercnv” R package based on large‐scale copy number variation (CNV) patterns inferred from single‐cell transcriptomic profiles. Immune and stromal cells were used as reference normal cells for CNV estimation. Cells exhibiting significant CNV alterations were classified as tumor cells.

### 2.2 Consensus Cluster Analysis of PRP

We identified 28 PRPs based on a systematic review of published literature, primarily according to the comprehensive review by Yu et al. [17], which summarized the core regulators and signaling pathways involved in pyroptosis. The selected PRPs included key components of canonical and noncanonical pyroptosis pathways, such as gasdermin family members, caspases, inflammasome‐associated proteins, and inflammatory cytokines. Detailed information for these PRPs is presented in Table S1. We performed univariate Cox regression analyses to identify prognosis‐related PRPs. We included variables with *p* < 0.05 in subsequent multivariate Cox regression analysis. We used the package “ConsensusClusterPlus” for consensus unsupervised cluster analysis to classify patients into different clusters based on PRP expression. We selected the optimal cluster number based on the cumulative distribution function (CDF) curve and cluster sample size stability.

### 2.3 DEP Identification and Functional Enrichment Analysis

We identified DEPs among different pyroptosis clusters using the Wilcoxon rank‐sum test. We defined DEPs as proteins with a fold change > 1.5 and an adjusted *p*‐value < 0.05. We performed Gene Ontology (GO) and Kyoto Encyclopedia of Genes and Genomes (KEGG) enrichment analyses using the R package “clusterProfiler.” We used these analyses to explore the biological functions and signaling pathways associated with the identified DEPs.

### 2.4 Construction of the PRP‐Related Prognostic Score

We first performed univariate Cox regression analysis to identify prognosis‐associated DEPs in LUAD. We then constructed a PRP‐related prognostic score using the training cohort. In brief, we applied least absolute shrinkage and selection operator (LASSO)‐Cox regression analysis using the R package “glmnet” to reduce overfitting. The trajectory of each independent variable was analyzed, and 10‐fold cross‐validation was used to build the model. We subsequently performed multivariate Cox regression analysis to establish the final prognostic model. The PRP score was calculated as follows: PRP score = *Σ* (Expi * coefi), where Expi represents the expression level of each protein and coefi represents the corresponding regression coefficient. Based on the median risk score, we divided patients into low‐risk (PRP score < median) and high‐risk (PRP score > median) groups. We evaluated OS using Kaplan–Meier survival analysis and the predictive performance using receiver operating characteristic (ROC) curve analysis. Similarly, the validation set was divided into low‐risk and high‐risk groups, each of which was subjected to Kaplan–Meier survival analysis and ROC curve analysis.

### 2.5 Drug Susceptibility Analysis

To compare drug sensitivity between the high‐risk and low‐risk groups, we estimated the half‐maximal inhibitory concentration (IC50) values of commonly used chemotherapeutic agents for LUAD using the “pRRophetic” software package.

### 2.6 TME and Immune Infiltration Analysis

We calculated stromal scores, immune scores, and ESTIMATE scores using the “ESTIMATE” algorithm. Additionally, we evaluated the proportions of 22 immune cell subtypes using the “CIBERSORT” algorithm. We further assessed immune cell infiltration levels using single‐sample gene set enrichment analysis (ssGSEA).

### 2.7 Tumor Immunogenicity and Intercellular Communication

We performed gene set variation analysis (GSVA) and gene set enrichment analysis (GSEA) using the R packages “GSVA” and “clusterProfiler.” The R package “CellChat” was employed to predict intercellular communication based on ligand‐receptor pairs between tumor cells and immune cells. We selected significantly altered ligand‐receptor pairs for further analysis.

### 2.8 Tissue Samples and Immunohistochemistry (IHC)

A total of four pairs of LUAD and adjacent nontumor tissue samples were collected from lung cancer patients who underwent tumor resection at Zhongshan Hospital of Fudan University in Shanghai, China. Informed consent was obtained from each relevant patient in accordance with the Declaration of Helsinki. All patients provided written informed consent. Ethical approval was obtained from the medical ethics committee of the hospital. Subsequently, the protein levels of the selected PRPs were verified by IHC experiments. All specimens were fixed in 10% formalin at room temperature, paraffin‐embedded, sectioned (4 μm), and then stained. Finally, the samples were sealed, observed with a light microscope, and photographed. The primary antibodies used were: OCIAD1 (proteintech, Cat No. 66698), SLC25A17 (proteintech, Cat No.67635), Syntaxin 2 (proteintech, Cat No.55033), PPAP2A (abcam, ab198280), WARS2 (Invitrogen, PA5‐67064). Although three pathologists were later found to score the sections and finally summarize the statistics. Data analyses were performed using GraphPad Prism 7.0 (La Jolla, California, USA).

### 2.9 Cell Culture and siRNA Transfection

We cultured A549 cell lines in Dulbecco's Modified Eagle Medium (DMEM) supplemented with 10% fetal bovine serum and 1% penicillin–streptomycin. We maintained cells at 37°C in a humidified incubator containing 5% CO2. We transfected cells with specific small interfering RNAs (siRNAs) targeting *OCIAD1* and *WARS2* using Lipofectamine 3000 reagent according to the manufacturer's instructions. We used scrambled siRNA as the negative control.

### 2.10 Quantitative Real‐Time PCR (qRT‐PCR)

We extracted total RNA using TRIzol reagent (Sigma). cDNA was synthesized using the FastKing RT Kit (Tiangen; Cat: KR116‐02) as per instructions. We performed quantitative real‐time PCR using SYBR Green Master Mix (Yeasen; Cat: 11203ES08) on the RT‐PCR 384 system (Bio‐Rad). The forward (F) and reverse (R) primer sequences for the target genes are listed in Table S9.

### 2.11 Immunoblotting

We extracted total protein from cells using RIPA lysis (Beyotime; Cat:P0013) buffer supplemented with protease inhibitors. Protein concentrations were determined by the BCA assay. We separated proteins by SDS‐PAGE and transferred them onto PVDF membranes. We blocked membranes with 5% nonfat milk and incubated them with primary antibodies overnight at 4°C. We then incubated membranes with HRP‐conjugated secondary antibodies. Protein bands were visualized using ECL and imaged with a ChemiDoc system. The antibodies included tubulin (Proteintech; Cat: 11224‐1‐AP), OCIAD1(proteintech, Cat No.66698), SLC25A17 (proteintech, Cat No.67635), Syntaxin 2 (proteintech, Cat No.55033), PPAP2A (abcam, ab198280), and WARS2 (Invitrogen, PA5‐67064).

### 2.12 Wound‐Healing Assay

We seeded transfected cells into six‐well plates and cultured them until reaching approximately 90% confluence. We generated a linear wound using a sterile pipette tip. We removed detached cells with phosphate‐buffered saline and cultured cells in serum‐free medium. We photographed wound areas at 0 and 24 h. We quantified cell migration by measuring wound closure areas.

### 2.13 Transwell Invasion Assay

We evaluated cell invasion ability using Transwell chambers coated with Matrigel (Corning, Cat: 356231). We seeded transfected cells into the upper chamber in serum‐free medium. We added medium containing 10% fetal bovine serum to the lower chamber. After incubation, we fixed invaded cells with paraformaldehyde and stained them with crystal violet. We counted invaded cells under a light microscope.

### 2.14 Statistical Analysis

We performed all statistical analyses using R software (version 4.1.3) and GraphPad Prism 7.0. We expressed continuous variables as mean ± standard deviation (SD). We compared differences between two groups using Student's *t*‐test or Wilcoxon rank‐sum test. We compared differences among multiple groups using one‐way analysis of variance (ANOVA) or Kruskal–Wallis test. We evaluated survival differences using Kaplan–Meier analysis and the log‐rank test. We assessed independent prognostic factors using Cox proportional hazards regression analysis. All experiments were independently repeated at least three times. We considered *p* < 0.05 statistically significant.

## 3 Results

### 3.1 Identification of Pyroptosis‐Related Clusters in LUAD

To comprehensively characterize the expression of PRPs during LUAD progression, we analyzed proteomic data and clinical information from 103 patients with LUAD obtained from a previously published cohort [14]. Univariate Cox regression and Kaplan–Meier survival analysis demonstrated that 28 PRPs were significantly associated with patient prognosis (Table S1 and Table S2). A threshold of *p* < 0.05 was used to identify prognosis‐related PRPs. We subsequently performed multivariate Cox regression analysis on six candidate prognostic proteins, among which CASP4 and GSDMB were identified as independent prognostic factors (Table 1).

**TABLE 1:** Multivariate Cox regression analysis of 2 PRGs associated with OS in LUAD patients.

Protein	HR	95%CI	*p*
PRKACA	0.32	0.16–0.64	0.0013
CASP4	1.6	1.1–2.4	0.0200

**FIGURE 1:**
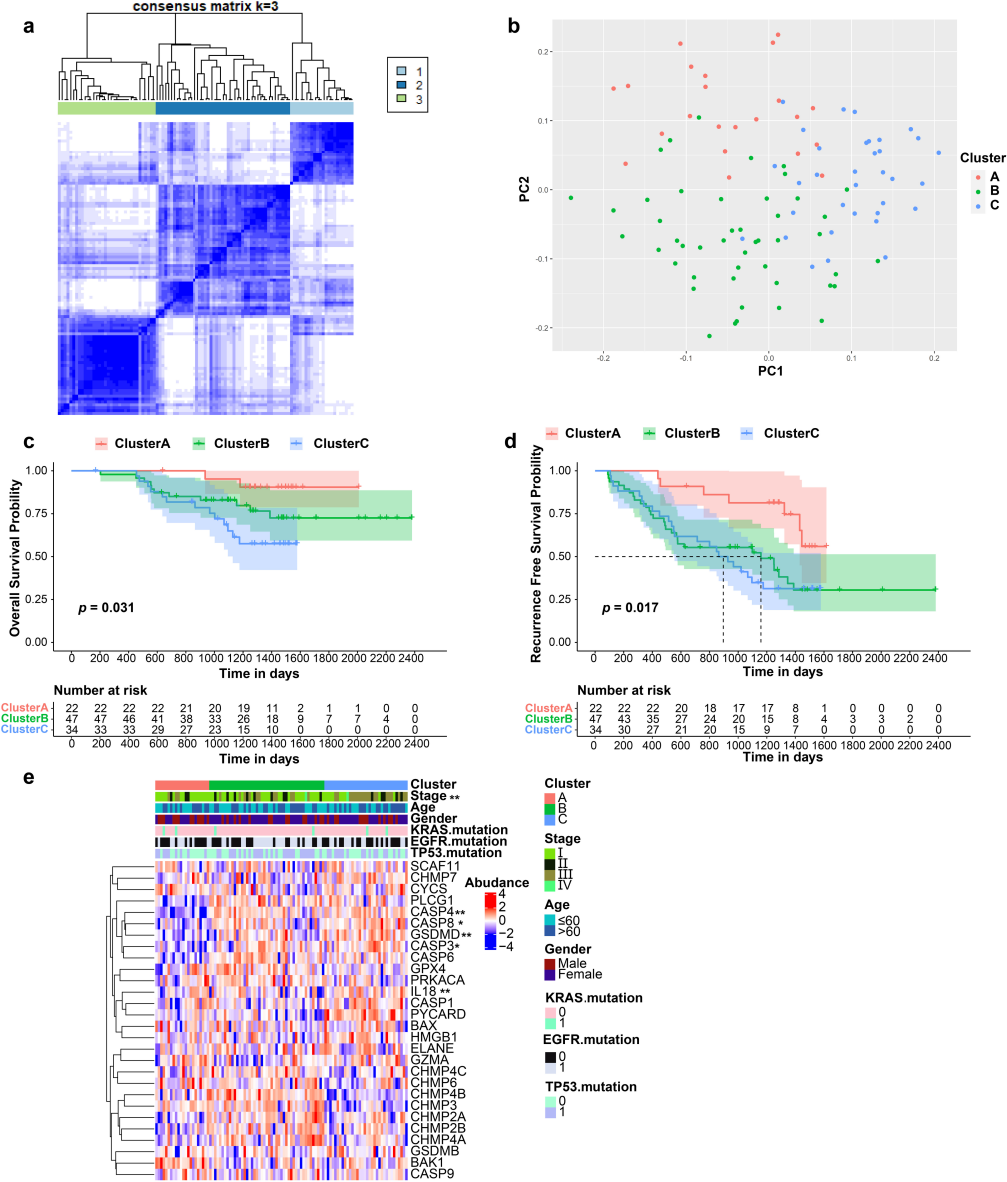
Identification of pyroptosis‐related clusters and clinicopathological characteristics in LUAD. (a) Consensus clustering heatmap of LUAD samples based on the expression profiles of PRPs (*k* = 3). (b) The PCA analysis of three PRP‐related clusters (c) Kaplan–Meier curves of OS for the three clusters (log‐rank test, *p* ≤ 0.05). (d) Kaplan–Meier curves of RFS for the three clusters (log‐rank test, *p* ≤ 0.05). (e) Heatmap of clinicopathological features and PRP expression patterns among the three clusters.

To further explore PRP expression patterns in LUAD, consensus clustering analysis was performed based on the expression profiles of 28 PRPs (Figure S1). The optimal clustering parameter was determined to be *k* = 3. Accordingly, the entire cohort was classified into Clusters A (*n* = 22), B (*n* = 47), and C (*n* = 34) (Figure 1a). PCA further confirmed clear transcriptional differences among the three clusters (Figure 1b).

Kaplan–Meier analysis revealed significant prognostic differences among the three clusters. Patients in Cluster A exhibited the most favorable OS (*p* = 0.031) and recurrence‐free survival (RFS) (*p* = 0.017). In contrast, patients in Cluster C showed the poorest RFS, whereas Cluster B demonstrated an intermediate prognosis (Figure 1c,d). Baseline clinicopathological characteristics of patients in the three PRP‐related subtypes are summarized in Table 2. Significant differences were also observed among the three clusters (Figure 1e). Cluster A was significantly associated with lower TNM stage (*p* ≤ 0.05). However, no significant differences were detected in TP53 mutations, KRAS mutations, or EGFR mutations among the clusters. Notably, Cluster A exhibited relatively lower expression of CASP4, CASP8, CAPS3, GSDMD, PYCARD, and IL‐18, whereas these proteins were highly expressed in Cluster C.

**TABLE 2:** Baseline clinicopathological characteristics of patients with LUAD according to PRP‐related clusters.

Characteristics	Overall (*n* = 103)	Cluster A (*n* = 22)	Cluster B (*n* = 47)	Cluster C (*n* = 34)	*p*
Age, years	59.11 (10.29)	58.95 (12.11)	58.13 (9.71)	60.56 (9.97)	0.580
Gender					0.135
Male	38 (36.9)	12 (54.5)	14 (29.8)	12 (35.3)	
Female	65 (63.1)	10 (45.5)	33 (70.2)	22 (64.7)	
Smoking status					0.747
Nonsmoker	77 (74.8)	16 (72.7)	34 (72.3)	27 (79.4)	
Smoker	26 (25.2)	6 (27.3)	13 (27.7)	7 (20.6)	
TNM stage					0.001
Stage I–II	68 (66.0)	19 (86.4)	35 (74.5)	14 (41.2)	
Stage III–IV	35 (34.0)	3 (13.6)	12 (25.5)	20 (58.8)	
TP53					0.431
WT	50 (48.5)	13 (59.1)	20 (42.6)	17 (50.0)	
Mut	53 (51.5)	9 (40.9)	27 (57.4)	17 (50.0)	
EGFR					0.328
WT	52 (50.5)	13 (59.1)	20 (42.6)	19 (55.9)	
Mut	51 (49.5)	9 (40.9)	27 (57.4)	15 (44.1)	
KRAS					0.726
WT	97 (94.2)	20 (90.9)	45 (95.7)	32 (94.1)	
Mut	6 (5.8)	2 (9.1)	2 (4.3)	2 (5.9)	

### 3.2 Functional Characteristics of Distinct Subtypes

**FIGURE 2:**
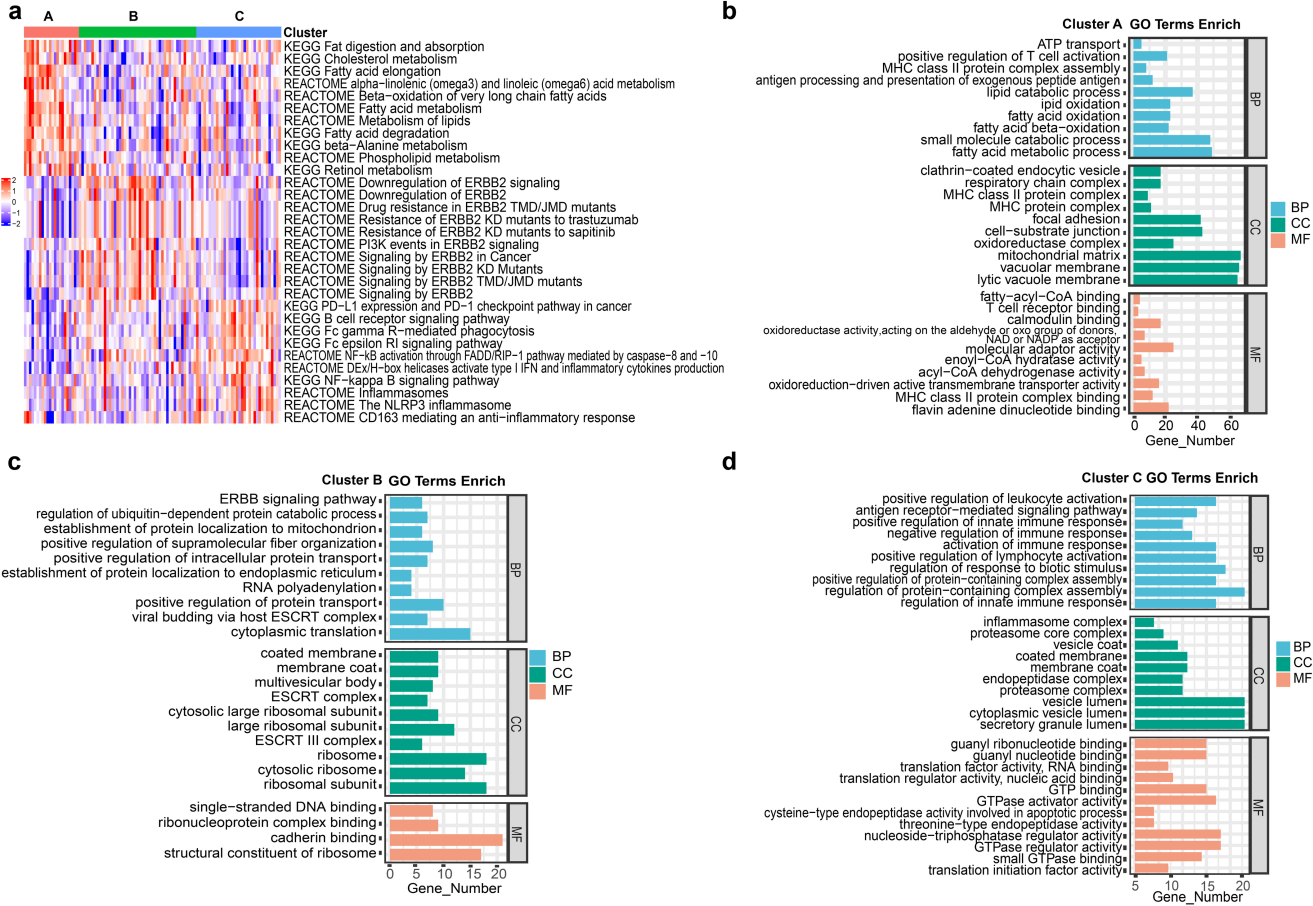
Characteristics of function in distinct clusters based on DEGs. (a) GSVA of biological pathways among three distinct clusters, in which red and blue represent activated and blue inhibited pathways, respectively. (b) GO enrichment analyses of DEPs in Cluster A. (c) GO enrichment analyses of DEPs in Cluster B. (d) GO enrichment analyses of DEPs in Cluster C.

To investigate the biological characteristics of the three PRP‐related subtypes, we performed GSVA analysis. The results demonstrated distinct functional enrichment patterns among the clusters (Figure 2a and Table S3). Cluster A was primarily enriched in lipid metabolism‐related pathways, including fatty acid elongation, fatty acid degradation, and multiple metabolic processes. Cluster B mainly showed enrichment in signal transduction pathways, particularly ERBB2‐related signaling pathways. In contrast, Cluster C demonstrated significant enrichment in immune activation‐related pathways, including the B‐cell receptor signaling pathway and NF‐κB signaling pathway. Gene Ontology (GO) enrichment analysis further supported these findings. Cluster A was mainly enriched in lipid metabolism and partially related to immune‐related biological functions (Figure 2b). Cluster B showed enrichment in signal transduction‐related functions and protein immune complex binding (Figure 2c). Cluster C was predominantly enriched in immune regulation‐associated biological processes (Figure 2d and Table S4). Notably, Cluster C showed strong correlations with multiple immune cell‐associated pathways, suggesting enhanced activation of pyroptosis within the tumor immune microenvironment. Based on these distinct functional characteristics, we defined Cluster A as the lipid metabolism subtype, Cluster B as the signal transduction subtype, and Cluster C as the immune activation subtype.

### 3.3 TME and Immunological Characteristics of Different Subtypes

**FIGURE 3:**
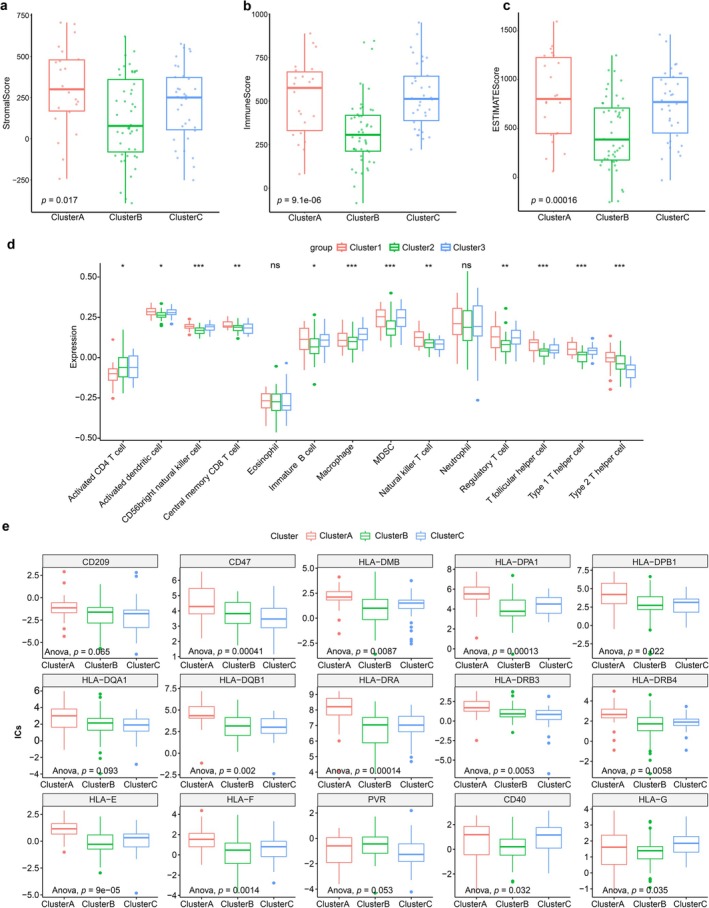
Tumor microenvironment and immune characteristics among three clusters. (a–c) Comparison of stromal score, immune score, and ESTIMATE score among the three clusters. (b) Distribution of infiltrating immune cell populations among the three LUAD clusters based on CIBERSORT analysis. (c) Expression of immune checkpoints in the three clusters.

To further investigate the role of PRPs in TME of LUAD, we evaluated stromal scores, immune scores, and ESTIMATE scores among the three subtypes using the ESTIMATE package (Figure 3a–c and Table S4). Higher stromal and immune scores indicated increased proportions of stromal cells and immune cells within the TME, whereas the ESTIMATE score was used to infer tumor purity. The results showed that patients in Cluster A had the highest TME scores. These findings suggested that Cluster A possessed a relatively complex and immune‐enriched microenvironment. We next applied the CIBERSORT algorithm to evaluate immune cell infiltration patterns across the three clusters (Figure 3d and Table S5). Significant differences in immune cell composition were observed among the clusters. Cluster A showed markedly increased infiltration of activated dendritic cells, CD56bright natural killer cells, central memory CD8 + T cells, immature B cells, myeloid‐derived suppressor cells, natural killer cells, regulatory T cells, follicular helper‐like T cells, type 1 helper T cells, and type 2 helper T cells compared with the other clusters. Additionally, Cluster C displayed the highest infiltration levels of activated CD4^+^ T cells and macrophages. Cluster B generally showed the lowest degree of immune cell infiltration. The results indicated distinct immune landscapes among the three PRP‐related clusters. Cluster A was characterized by increased infiltration of immunoregulatory and immunosuppressive cell populations, whereas Cluster C exhibited features of enhanced immune activation. We further analyzed the expression of major histocompatibility complex (MHC)‐related immune genes. Patients in cluster A showed significantly higher expression of multiple antigen presentation‐associated genes, including HLA‐DPB1, HLA‐DQA1, HLA‐DQB1, HLA‐DRA, HLA‐DRB3, HLA‐DRB4, HLA‐E, and HLA‐F (Figure 3e). The elevated expression of these genes may facilitate tumor antigen presentation and contribute to the favorable prognosis observed in Cluster A.

### 3.4 Construction and Validation of PRP‐Related Prognostic Score

We constructed the PRP‐related prognostic score based on identified among the three proteomic clusters. First, univariate Cox regression analysis was performed on the DEPs to identify prognosis‐associated proteins. We subsequently conducted LASSO regression analysis and multivariate Cox regression analysis to select the optional prognostic features. After LASSO regression analysis, 16 candidate proteins with the minimum partial likelihood deviance remained (Figure S1a,b). Multivariate Cox regression analysis was then performed using the Akaike information criterion (AIC) values. Finally, six proteins, including PPAP2A, OCIAD1, WARS2, BRD1, STX2, and SLC25A17, were selected to establish the prognostic model. Among these proteins, PPAP2A and WARS2 were identified as unfavorable prognostic factors, whereas OCIAD1, BRD1, STX2, and SLC25A17 were identified as favorable prognostic factors. The PRP score was calculated using the following formula:

$$\begin{array}{c} \text{Risk Score}=\left(0.52559^{*}\text{PPAP}2A\;\text{expression}\right)\\ \;+\left(-0.75506^{*}\text{OCIAD1 expression}\right)\\ \;+\left(0.83785^{*}\text{WARS2 expression}\right)\\ \;+\left(-0.35816^{*}\text{BRD1 expression}\right)\\ \;+\left(-0.40693^{*}\;\text{STX2 expression}\right)\\ \;+\left(-0.43101^{*}\;\mathrm{SLC}2\text{5A}17\;\text{expression}\right). \end{array}$$

**FIGURE 4:**
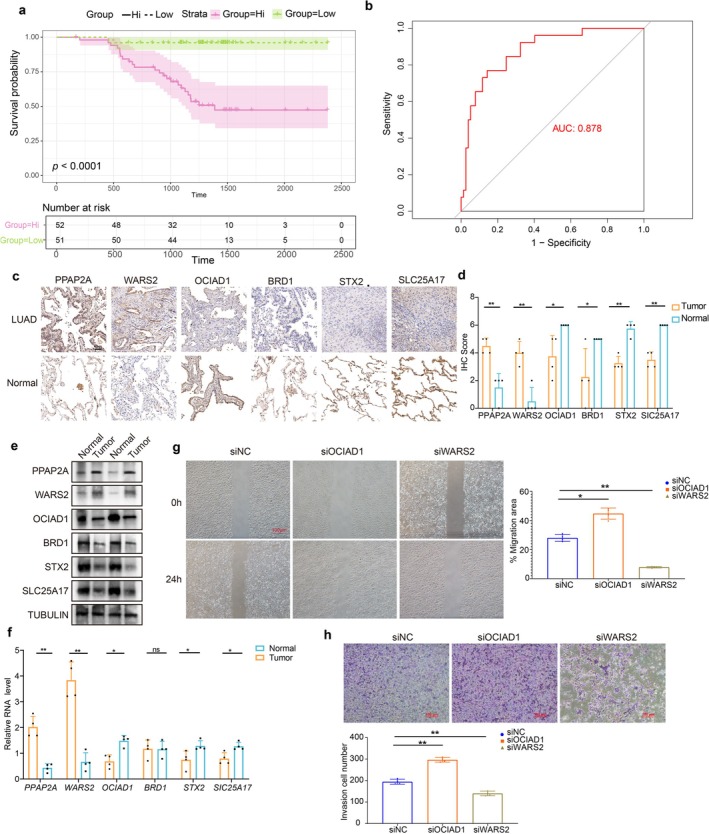
Construction of the PRP score in the training set. (a) Kaplan–Meier analysis of the OS for the high‐risk and low‐risk group. (b) ROC curve analysis of the predictive performance of the PRP score. (c) Representative IHC staining of six selected proteins in LUAD and adjacent nontumor tissues. (d) Quantitative analysis of immunohistochemical staining scores for the six selected PRPs. (e) Western blot analysis of selected PRPs in LUAD samples. (f) Quantitative real‐time PCR analysis of selected PRPs in LUAD samples. (g) Wound‐healing assay showing migration abilities of A549 cells after transfection with siNC, siOCIAD1, or siWARS2. (h) Transwell invasion assay showing invasion abilities of A549 cells after transfection with siNC, siOCIAD1, or siWARS2.

Patients were divided into low‐risk (*n* = 51) and high‐risk (*n* = 52) groups according to the median PRP score (Table S6). Kaplan–Meier survival analysis indicated that patients in the low‐risk group exhibited significantly better overall survival compared to those in high‐risk group (*p* < 0.001) (Figure 4a). ROC curve analysis further showed that the PRP score had strong predictive performance, with an area under the curve (AUC) value of 0.878 for 5‐year survival prediction (Figure 4b).

To further validate the prognostic performance of the PRP score, we analyzed two independent external cohorts, including a proteomics dataset16 and a transcriptomic dataset (GSE41721) [16]. Patients in both validation cohorts were classified into low‐risk and high‐risk groups using the same scoring formula. Consistent with the training cohort, patients in the low‐risk group showed significantly more favorable survival outcomes than those in the high‐risk group (*p* < 0.01) (Figure S2a–d). ROC curve analysis also demonstrated satisfactory predictive accuracy in the validation cohorts. Immunohistochemical staining demonstrated that PPAP2A and WARS2 were significantly upregulated in tumor tissues, whereas OCIAD1, BRD1, STX2, and SLC25A17 were downregulated compared with adjacent normal tissues (Figure 4c,d and Table S7). These expression patterns were consistent with the prognostic model. Western blot analysis further confirmed the differential expression of the five selected proteins in LUAD samples, and the expression trends were consistent with the PRP scoring system (Figure 4e). Quantitative real‐time PCR analysis produced similar results at the transcriptional level (Figure 4f).

To further evaluate the biological functions of PRPs in LUAD progression, we performed loss‐of‐function experiments in A549 cells. Wound‐healing and Transwell invasion assays showed that OCIAD1 knockdown promoted cell migration and invasion, whereas WARS2 knockdown suppressed these malignant phenotypes (Figure 4g,h). These findings were consistent with the prognostic roles identified in the PRP score model.

### 3.5 Identification of Subtypes in scRNA Data

**FIGURE 5:**
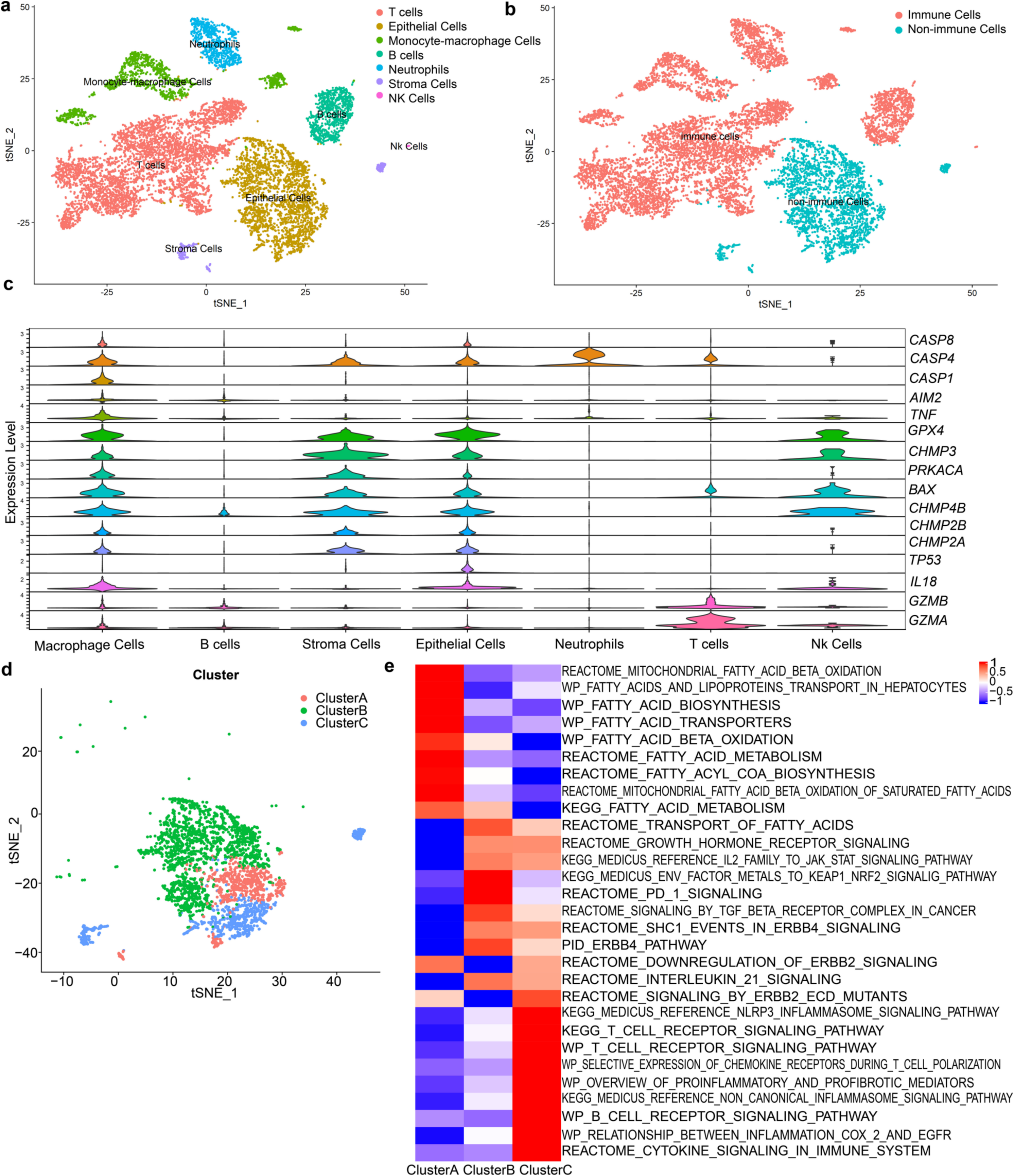
ScRNA sequencing analysis and functional validation of PRPs in LUAD. (a,b) UMAP distribution of cell populations in scRNA‐seq analysis. (c) Expression patterns of pyroptosis‐related genes across different cell populations. (d) The cluster model in sc RNA seq analysis. (e) KEGG enrichment analyses of DEGs among three clusters.

We further performed scRNA‐seq analysis of nine LUAD samples to characterize the cellular composition of the TME (Figure 5a,b). The expression profiles of pyroptosis‐related genes (PRGs) were subsequently analyzed across different cell populations. Most PRGs showed relatively high expression in macrophages and tumor cells compared with other cells (Figure 5c). To correlate the scRNA‐seq data with the proteomics‐based subtypes, we performed correlation analysis between nonimmune cells and the three clusters. Based on the correlation coefficients, nonimmune cells were classified into three corresponding subtypes (Figure 5d). We next performed pathway activity analysis for each cell subtype using AUCell scoring (Figure 5e, Table S8). Consistent with the proteomic findings, Cluster A was mainly enriched in lipid metabolism‐related pathways, including fatty acid synthesis, transport, and beta‐oxidation. Cluster B predominantly showed enrichment in signaling transduction‐related pathways, such as the ERBB4 and PD1 signaling pathways. In contrast, Cluster C was significantly enriched in immune activation‐associated pathways, including T‐cell receptor, B‐cell receptor, and cytokine signaling pathways. These findings demonstrated strong concordance between the proteomic and single‐cell transcriptomic analyses.

### 3.6 Distinct Intercellular Communication Networks Among the Three Clusters

**FIGURE 6:**
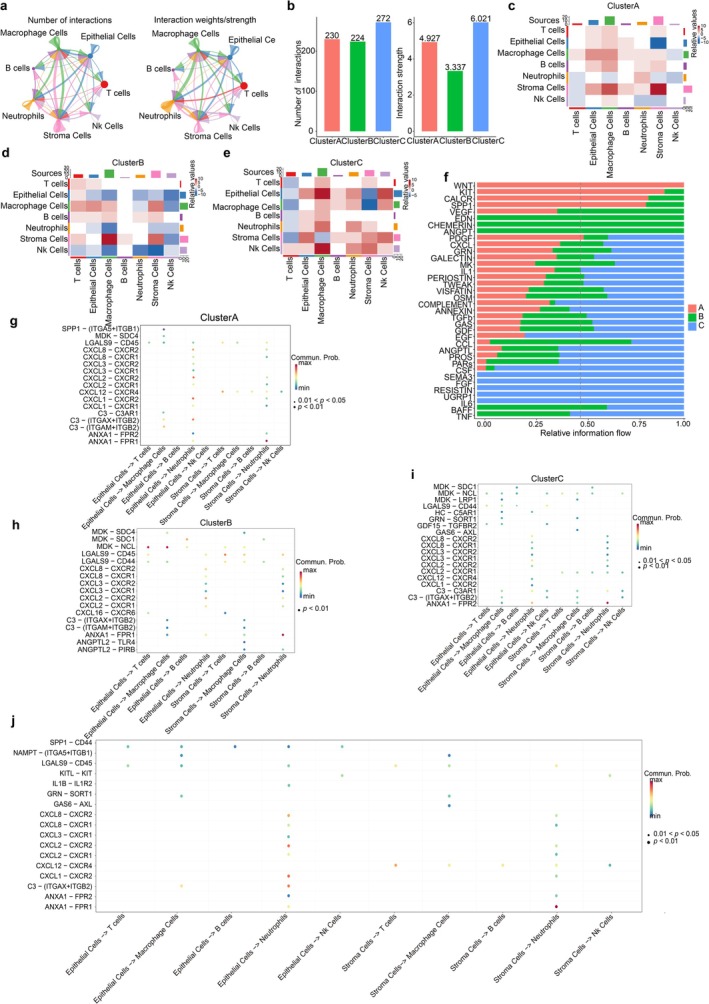
Intercellular communication networks among three clusters. (a,b) Cellular interaction network among major cell populations in LUAD samples. (c–e) Heatmaps of the cellular interaction scores among different clusters. (f) Signaling pathway network analysis among three clusters. (g) Ligand‐receptor interaction networks between tumor cells and immune cells. (h–j) Intercellular communication networks in different clusters.

To investigate the interactions within the TME, we analyzed communication networks between tumor cells and immune cells using CellChat analysis (Figure 6a,b). Extensive interactions were observed between epithelial cells and mesenchymal cells, as well as between T cells and macrophages. Among the three subtypes, Cluster C exhibited the strongest and most diverse intercellular communication network. Distinct communication patterns were identified among the three subtypes. Cluster A exhibited relatively fewer interactions between epithelial cells and mesenchymal cells, whereas interactions involving macrophages and mesenchymal cells were more prominent (Figure 6c). In Cluster B, macrophages and mesenchymal cells had the highest number of interactions, but the number of interactions between immune cells was low; also, it showed frequent interactions; however, interactions among immune cells were relatively limited (Figure 6d). In contrast, Cluster C displayed extensive interactions between immune cells and epithelial or mesenchymal cells (Figure 6e). Pathway enrichment analysis further revealed subtype‐specific signaling networks (Figure 6f). SEMA3, FGF, resistin, UGRP1, IL‐6, and BAFF signaling pathways were specifically enriched in Cluster C. Chemerin and ANGPT signaling pathways were predominantly enriched in Cluster B, whereas WNT signaling was mainly enriched in Cluster A. Further analysis of ligand‐receptor interactions suggested the presence of a complex communication network involving MDK, ANGPTL2, and chemokine‐related signaling pathways (Figure 6g). Notably, epithelial cells expressed high levels of MDK ligands that potentially interacted with neuronal ceroid lipofuscinoses (NCL), SP1‐LDL receptor‐related protein 1 (LRP1), and syndecan 1 (SDC1) receptors expressed by T cells, macrophages, and B cells. Different dominant signaling pathways were observed across the three subtypes (Figure 6h–j). Cluster A exhibited relatively strong activation of chemokine signaling, particularly between epithelial cells and neutrophils, whereas MDK and osteopontin (SPP1) signaling activities were relatively weak. In contrast, Cluster B cells exhibited stronger MDK signaling activity. In addition, all three subtypes demonstrated high expression of the ANXA1‐FPR1 signaling axis, suggesting its potential importance in LUAD progression and therapeutic response.

### 3.7 Immune Checkpoint Characteristics and Drug Susceptibility Analysis

**FIGURE 7:**
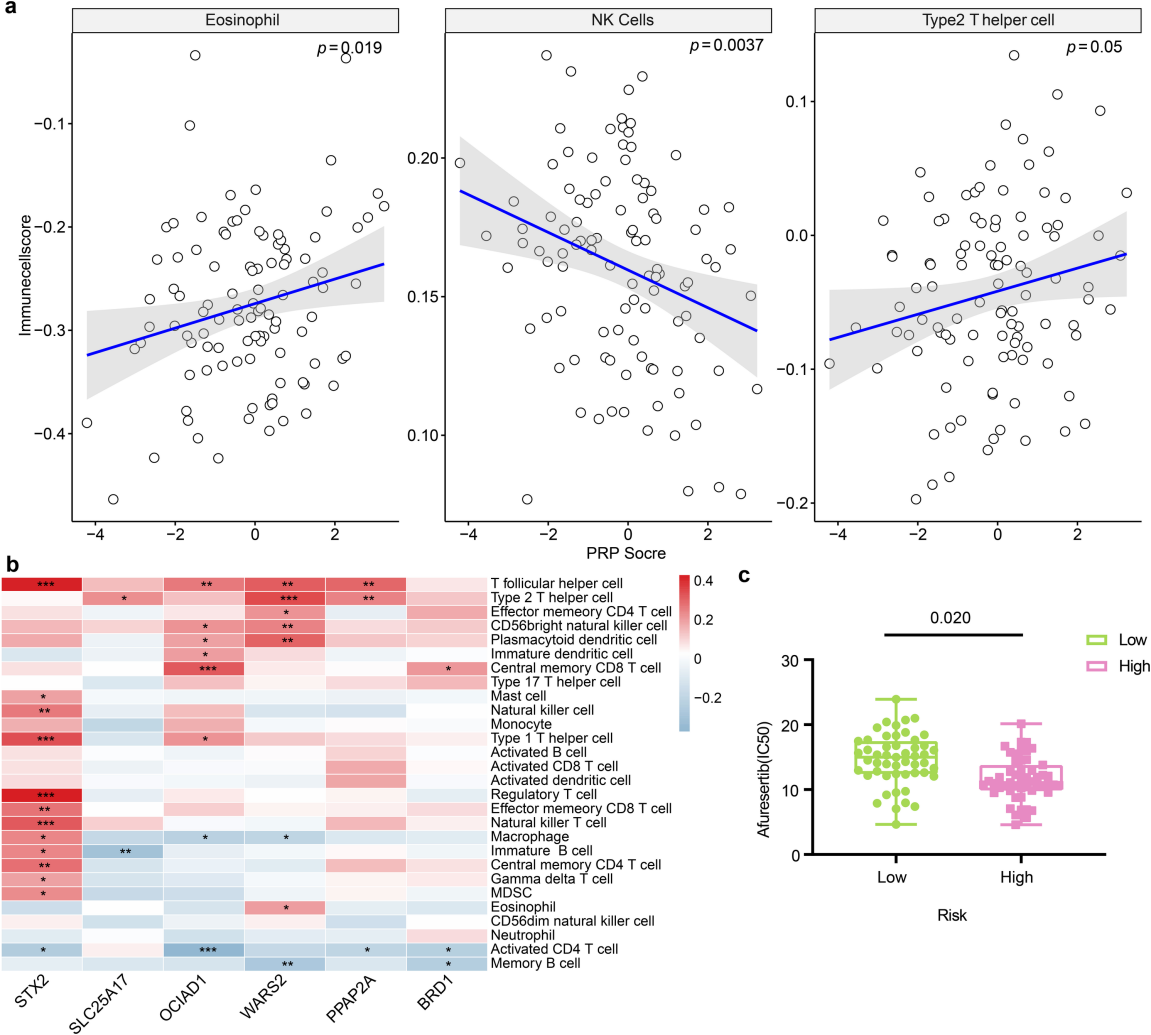
Comprehensive analysis of the PRP score in LUAD. (a) Correlation analysis between PRP score and immune cell infiltration levels. (b) Correlations analysis between immune cell abundance and the six proteins included in the prognostic model. (c) Drug sensitivity analysis between the high‐risk and low‐risk groups.

We further evaluated the relationship between the PRP score and immune cell infiltration using the CIBERSORT algorithm (Figure 7a). Correlation analysis demonstrated that the PRP score was positively associated with eosinophils and type 2 helper T cells, whereas it was negatively associated with activated natural killer cells. We also observed significant correlations between immune cell infiltration and six proteins. Among these proteins, STX2 showed the strongest associations with multiple immune cell populations (Figure 7b). To evaluate potential therapeutic responses, we analyzed the sensitivity of LUAD patients to commonly used chemotherapeutic agents. Patients in the low‐risk group demonstrated higher sensitivity to AKT‐targeted agents compared with those in the high‐risk group (Figure 7c). This finding may partially explain the favorable prognosis observed in the low‐risk subgroup.

## 4 Discussion

Pyroptosis is a form of programmed cell death mediated by gasdermin family proteins following caspase activation. Increasing evidence has demonstrated that pyroptosis plays critical roles in innate immunity, tumor progression, and antitumor responses [18, 19]. Recent studies have also suggested that pyroptosis may serve as a promising therapeutic target in multiple diseases, including infectious diseases and cancer [19]. However, the biological significance of PRPs in LUAD remains incompletely understood, particularly at the proteomic and TME levels. Most previous studies have primarily focused on the transcriptomic expression of PRGs or on individual immune cell populations. In contrast, the global protein‐level alterations of PRPs and their associations with TME heterogeneity have not been systematically investigated. We comprehensively characterized PRP expression patterns in LUAD using proteomic data and scRNA‐seq data. We identified three distinct clusters based on 28 PRPs, including the lipid metabolism subtype, signal transduction subtype, and immune activation subtype. Significant differences in prognosis, biological functions, immune infiltration, and intercellular communication were observed among these subtypes. Patients in Cluster A exhibited the best OS and RFS, whereas patients in Cluster C showed relatively poor prognosis. Functional enrichment analysis demonstrated that Cluster A was mainly associated with lipid metabolism pathways, Cluster B was enriched in signal transduction pathways, and Cluster C was characterized by immune activation‐related pathways. These findings suggested that PRPs may participate in LUAD progression through distinct biological programs.

The relationship between lipid metabolism and the prognosis of LUAD has attracted increasing attention in recent years [20]. Previous studies demonstrated that dysregulation of glycerophospholipid metabolism, fatty acid metabolism, and arachidonic acid signaling pathways is closely associated with LUAD prognosis [21]. In addition, lipid metabolism‐related molecules such as lysophosphatidylcholine were reported to suppress tumor proliferation [22]. Our findings further support the potential protective role of lipid metabolism‐associated pathways in LUAD.

The ERBB pathway also plays a pivotal role in LUAD, and a number of drugs targeting this pathway have been initially employed [23, 24]. In the present study, Cluster B demonstrated enrichment in multiple signaling transduction pathways, indicating that PRP‐related alterations may contribute to tumor progression through oncogenic signaling regulation. Cluster C was predominantly enriched in immune activation‐related pathways and showed extensive immune cell infiltration. Previous studies reported that increased infiltration of activated immune cells, including macrophages and activated T cells, may be associated with poor prognosis in LUAD [25, 26]. Consistent with these findings, Cluster C exhibited high levels of macrophage infiltration and enhanced immune‐related signaling activity. Although immune activation is generally considered beneficial for antitumor responses, persistent inflammatory activation may also promote tumor progression and immunosuppressive remodeling of the TME.

The scRNA‐seq analysis further validated the molecular characteristics identified in the proteomic analysis. Similar pathway enrichment patterns were observed between proteomic data and scRNA‐seq data. In addition, distinct intercellular communication patterns were identified among the three subtypes. Cluster C exhibited the strongest and most complex communication network, particularly among epithelial cells, macrophages, and T cells. Further ligand‐receptor interaction analysis identified MDK‐related signaling as a major communication pathway within the TME. Previous studies demonstrated that MDK expression progressively increases during tumor progression and contributes to cancer development [27, 28]. In our study, epithelial cells highly expressed MDK ligands that potentially interacted with NCL, LRP1, and SDC1 receptors on immune cells. These findings suggest that MDK‐mediated signaling may participate in immune remodeling in LUAD. We also observed high expression of the ANXA1‐FDP1 signaling across all three clusters. Notably, MDX‐124, a novel ANXA1‐targeting agent, has recently shown potential therapeutic value in lung cancer [29]. Therefore, the ANXA1‐FPR1 axis identified in our study may provide additional therapeutic insights for LUAD treatment.

To further evaluate the clinical significance of PRPs, we constructed and validated a PRP‐related prognostic score based on six proteins. This model demonstrated strong predictive performance in both the training cohort and external validation cohorts. Patients with different PRP scores exhibited distinct survival outcomes, immune infiltration patterns, and drug sensitivity profiles. Furthermore, immunohistochemistry, Western blotting, and qRT‐PCR experiments validated the expression patterns of selected PRPs in LUAD tissues. Functional experiments further supported the biological roles of PRPs in LUAD progression. OCIAD1 knockdown promoted migration and invasion of LUAD cells, whereas WARS2 knockdown suppressed these malignant phenotypes. These findings were consistent with the prognostic characteristics identified in the PRP scoring model and further strengthened the biological relevance of our results.

Several limitations should also be acknowledged. First, most analyses in this study were based on retrospective public datasets, which may introduce potential selection bias. Second, although we performed preliminary functional validation experiments, the molecular mechanisms underlying PRP‐mediated regulation of tumor immunity and pyroptosis remain incompletely understood. Additional in vivo and mechanistic studies are required to further validate our findings.

In conclusion, our study characterized the proteomic and scRNA‐seq landscape of PRPs in LUAD. We identified three distinct PRP‐related molecular subtypes with unique biological and immune characteristics. We also established a robust prognostic model with potential clinical utility. These findings improve the current understanding of pyroptosis in LUAD and may provide novel strategies for prognostic evaluation and personalized immunotherapy.

## Author Contributions

T.W., Y.W., and C.Z. designed the research, interpreted the data, and wrote the manuscript. T.W. and Y.W. shared the cofirst authorship; order of cofirst authors was determined based on their contribution to the study. C.Z., J.X., and J.Y. helped to develop the experiments. X.C., C.L., and Y.S. supervised the study, provided scientific insight, and reviewed and edited the manuscript.

## Funding

This study was supported by the National Natural Science Foundation of China (82570107, 82130001, 82500110, 82203517, 82200089, 82400103), Shanghai Municipal Science and Technology Major Project (ZD2021CY001), Noncommunicable Chronic Diseases‐National Science and Technology Major Project (2024ZD052930), R&D Program of Guangzhou National Laboratory (GZNL2024A02003), Zhongshan Hospital, Fudan University (ZY2024‐001, ZSLCYJ202319), China Postdoctoral Science Foundation (2024M750545, 2024T170162), and Outstanding Resident Clinical Postdoctoral Program of Zhongshan Hospital Affiliated to Fudan University (2025ZYYS‐004).

## Ethics Statement

The Ethics Committee of Zhongshan Hospital, Fudan University, gave its approval to the project (ethics approval no. B2021‐128). All participants provided written informed consent to participate in the study and for their data to be published.

## Conflicts of Interest

The authors declared no conflicts of interest.

## Supporting information


**Figure S1:** Unsupervised clustering of pyroptosis‐related genes and Consensus matrix heatmaps for *k = 2–4*.
**Figure S2:** Validation of PRP score in testing sets. (a) KM analysis of the OS between the two groups in proteomics dataset (b) ROC curves to predict the sensitivity and specificity of overall survival according to the PRP score. (c) KM analysis of the OS between the two groups in transcriptomics dataset (b) ROC curves to predict the sensitivity and specificity of overall survival according to the PRP score.

**Table S1:** Pyroptosis‐related protein.
**Table S2:** The prognostic values of 28 PRPs in patients with LUAD.
**Table S3:** The activation states of biological pathways in distinct clusters by GSVA enrichment analysis.
**Table S4:** Immune scores of LUAD patients.
**Table S5:** Relative fractions of tumor‐infiltrating immune cells of LUAD patients by the CIBERSORT algorithm.
**Table S6:** The PRP scores of LUAD patients.
**Table S7:** Functional annotation of the differentially expressed proteins between three clusters.
**Table S8:** The activation states of biological pathways in distinct clusters by AUCell enrichment analysis.

## Data Availability

The datasets used and/or analyzed during the current study are available from the corresponding author on reasonable request.
