# Supplementary material for: Integrated Proteomic and scRNA‐Seq Analysis Reveals Pyroptosis‐Related Subtypes in Lung Adenocarcinoma
Source: Clin Respir J. 2026 Jul 5;20(7):e70210. doi: 10.1111/crj.70210 (PMC13333213; doi:10.1111/crj.70210)
Supplement: Supplementary file 1 — Figure S1: Unsupervised clustering of pyroptosis‐related genes and Consensus matrix heatmaps for k = 2–4. Figure S2: Validation of PRP score in testing sets. (a) KM analysis of the OS between the two groups in proteomics dataset (b) ROC curves to predict the sensitivity and specificity of overall survival according to the PRP score. (c) KM analysis of the OS between the two groups in transcriptomics dataset (b) ROC curves to predict the sensitivity and specificity of overall survival according to the PRP score. [file CRJ-20-e70210-s001.docx]

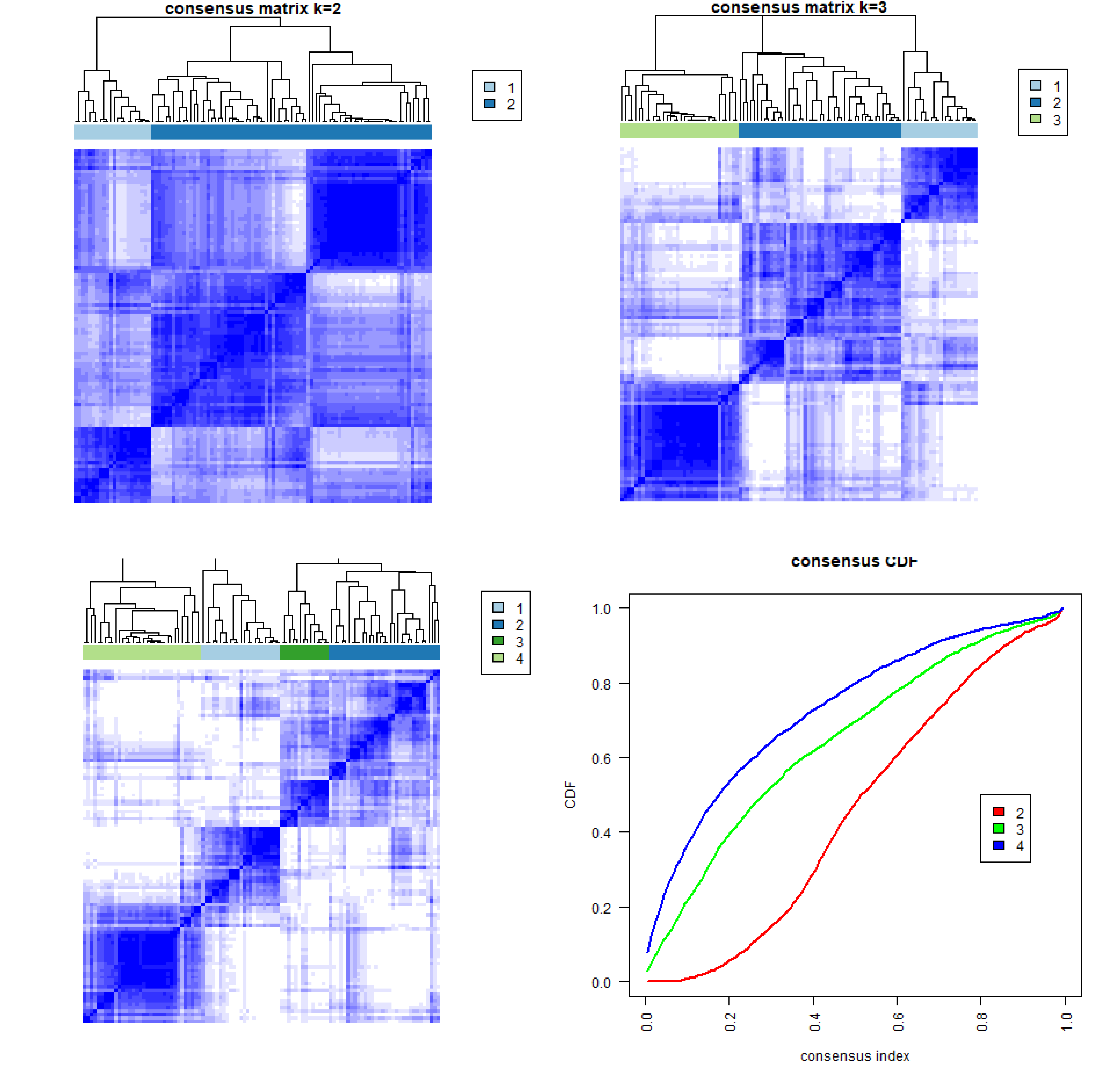
Figure S1. Unsupervised clustering of pyroptosis-related genes and Consensus matrix heatmaps for k = 2-4.


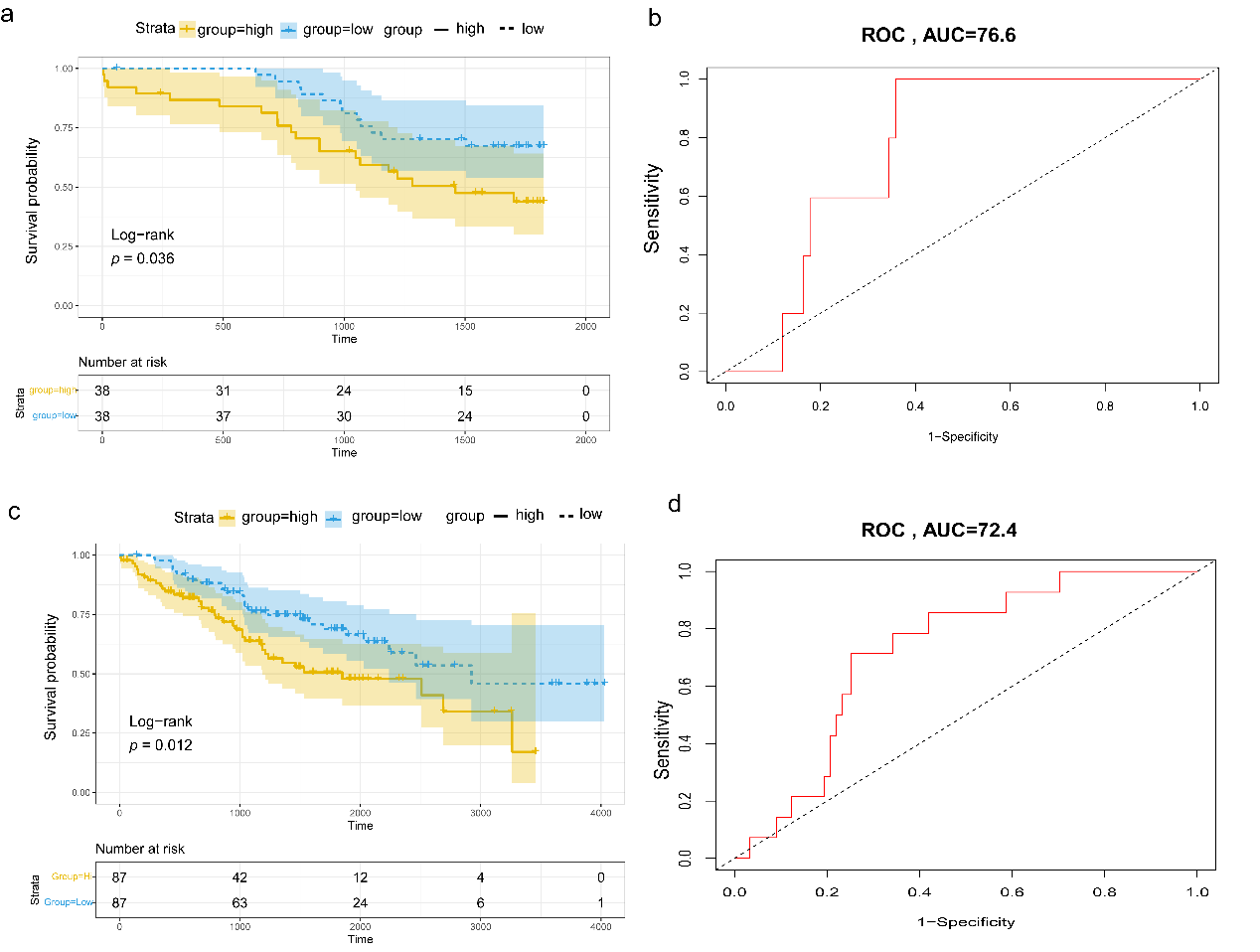


**Figure S2.** Validation of PRP score in testing sets. (a) KM analysis of the OS between the two groups in proteomics dataset (b) ROC curves to predict the sensitivity and specificity of overall survival according to the PRP score. (c) KM analysis of the OS between the two groups in transcriptomics dataset (b) ROC curves to predict the sensitivity and specificity of overall survival according to the PRP score.
